# Surgical Technique and Preliminary Outcomes of Double-Level Osteotomy for Valgus Deformity

**DOI:** 10.1177/23259671241252167

**Published:** 2024-06-03

**Authors:** Umito Kuwashima, Shuntaro Nejima, Marco Maiotti, Marc-Daniel Ahrend, Steffen Schröter

**Affiliations:** * Department of Orthopaedic Surgery, Tokyo Women's Medical University, Tokyo, Japan; † Department of Orthopaedic Surgery, Yokohama City University School of Medicine, Yokohama, Japan; ‡ Osteotomie Komitee der Deutschen Knie Gesellschaft, München, Germany; § Shoulder Unit Villa Stuart Clinic (Rome)–Orthopedics, Rome, Italy; ‖ Department of Traumatology and Reconstructive Surgery, BG Klinik Tübingen, Eberhard Karls University of Tübingen, Tübingen, Germany; # Department of Orthopaedics and Traumatology, Diakonie Klinikum, Jung-Stilling, Krankenhaus, Siegen, Germany; Investigation performed at BG Unfallklinik Tu « bingen, Eberhard Karls University of Tu « bingen, Tu « bingen, Germany

**Keywords:** double-level osteotomy, valgus knee, osteoarthritis

## Abstract

**Background:**

Data are limited regarding the surgical technique or outcomes of double-level osteotomy (DLO) combining medial closing-wedge distal femoral osteotomy and medial closing-wedge high tibial osteotomy in patients with moderate-to-severe valgus deformity.

**Purpose/Hypothesis:**

To describe the surgical technique and assess the short-term outcomes and surgical accuracy of DLO in patients with a moderate or severe valgus deformity. It was hypothesized that this technique would result in good clinical outcomes with precise deformity correction.

**Study Design:**

Case series; Level of evidence, 4.

**Methods:**

Eight patients (mean age, 44.2 ± 10.9 years) with a moderate or severe valgus knee treated with DLO (9 knees; mechanical tibiofemoral angle [mTFA], 10.3°± 3.5°) were included. The mean follow-up was 25.1 ± 11.1 months. Preoperative to postoperative changes in radiographic parameters (mTFA, mechanical lateral distal femoral angle, mechanical medial proximal tibial angle, joint line convergence angle) and clinical scores (Hospital for Special Surgery score, Oxford Knee Score, Lysholm score) were assessed. Surgical accuracy was calculated by subtracting the achieved postoperative correction from the preoperatively planned targeted correction.

**Results:**

The mTFA changed significantly from 10.3°± 3.5° preoperatively to –1.8°± 3.4° postoperatively (*P* < .001); the mechanical lateral distal femoral angle and mechanical medial proximal tibial angle changed significantly by 5.1°± 2.7° and 5.9°± 2.2°, respectively (*P* < .001 for both); and the posterior distal femoral angle decreased significantly from 85.9°± 3.1° to 84.2°± 2.4° (*P* < .01). There was no significant difference between pre- and postoperative joint line convergence angles (3.3°± 2.3° to 2.6°± 2.1°). The accuracy of the correction was high: the mTFA achieved postoperatively differed from the mTFA planned preoperatively by a mean of 2.7°± 1.9° (range, 0.6°-6.6°). Significant pre- to postoperative improvement was seen for all outcome scores (Hospital for Special Surgery, from 67 ± 11 to 93 ± 4; Oxford Knee Score, from 29 ± 7 to 43 ± 3; Lysholm, from 41 ± 24 to 89 ± 8; *P* < .001 for all).

**Conclusion:**

High surgical accuracy was achieved, and patients who underwent varus DLO for valgus knees showed improved knee function at short-term follow-up. Varus DLO can be a surgical option to restore the optimal alignment and joint line obliquity in patients with moderate or severe valgus malalignment.

In recent years, the outcome of osteotomies around the knee has improved because of technical and surgical developments.^27,34,50^ Osteotomy is a well-established treatment option for osteoarthritis (OA) in both valgus and varus knees.^24,37,42,47^ Correct planning is the key to a successful osteotomy, and a variety of approaches can be used to achieve a good result. Digital planning leads to reliable accuracy^44^ independent of the software used.^13,14,45^

In severe varus deformity, single-level osteotomy has proven to be inappropriate because it corrects the limb malalignment without maintaining neutral joint line obliquity.^4,19,39-41^ Correction of varus in combined deformities with a single-level osteotomy has inferior long-term results compared with double-level osteotomy (DLO) addressing the deformity of both bones.^52^ The increase of shear forces after an overcorrection at the proximal tibia is likely the reason for this finding.^33^ A DLO can precisely correct the deformity and retain the neutral joint line obliquity. Babis et al^4^ reported on the first encouraging results, and other working groups using a minimally invasive approach reported on a high level of accuracy and good clinical results.^2,34^

There are fewer publications on osteotomy for valgus deformities. Most studies describe the surgical technique of distal femoral osteotomy (DFO)^16,28,55^; little research has been performed on closing-wedge high tibial osteotomy (HTO) in valgus knees.^9-11,17,22,36,48^ The valgus deformity has to be corrected at the location where the deformity exists.^9^ Eberbach et al^12^ showed that a valgus deformity can be located in the femur (23.6%), tibia (41.0%), or femur and tibia combined (26.9%). Saragaglia and Chedal-Bornu^38^ reported on 29 cases of valgus deformity. In 5 of these cases, a DLO was performed. However, they used a medial closing-wedge HTO and a lateral opening-wedge DFO.

Data are limited regarding the surgical technique and outcome of DLO combining medial closing-wedge DFO and medial closing-wedge HTO in patients with severe valgus deformity. Thus, the purpose of this study was to describe the surgical technique and assess the short-term outcomes as well as the surgical accuracy of DLO in patients with moderate or severe valgus deformity. We hypothesized that the technique would result in good clinical outcomes with precise deformity correction.

## Methods

### Study Patients

This retrospective case series was approved by our institutional ethics commission, and informed consent was obtained from all included patients. Inclusion criteria were combined valgus deformity of the femur and tibia (mechanical medial proximal tibial angle [mMPTA] >90° and mechanical lateral distal femoral angle [mLDFA] <85°, or simulation of a single osteotomy would result in a new deformity), lateral compartment OA, age ≥18 years, the ability to walk >500 meters, the ability to perform moderate activities of daily living, and knee joint pain resistant to nonoperative treatment. Exclusion criteria were lack of patient consent, age <18 years old, severe disability combined with ability to walk <500 meters, and single-bone deformities.

Between July 2013 and October 2015, 12 DLO procedures for valgus knee were performed in 11 patients. Eight patients (9 knees; 9 DLOs) with postoperative follow-up data were included in the study. There were 5 women and 3 men with a mean age of 44.2 ± 10.9 years (range, 31-60 years). The mean follow-up was 25.1 ± 11.1 months (range, 12.9-40.0 months). Patient characteristics are summarized in Table 1.

**Table 1:** Demographic Data of the Study Cohort (9 Knees, 8 Patients)^*a*^

Variable	Value
Age preoperatively, y	44.2 ± 10.9 (31-60)
Sex, male/female, n	3/6
Body mass index, kg/m^2^	32.5 ± 4.9 (25.8-40.1)
Kellgren-Lawrence OA grade, 1/2/3/4, n	2/1/0/6
Follow-up period, mo	25.1 ± 11.1 (12.9-40.0)

a Data are reported as mean ± SD (range) unless otherwise indicated. OA, osteoarthritis.

**Figure 1.**
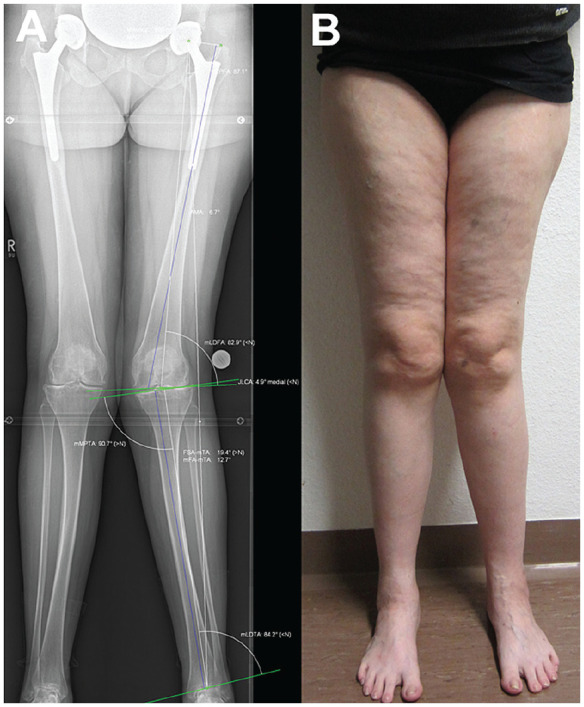
Case example: 55-year-old female patient with lateral knee pain. (A) Preoperative radiograph (deformity analysis: mechanical tibiofemoral angle [mTFA], 12.7°; mechanical lateral distal femoral angle [mLDFA], 82.9°; mechanical medial proximal tibial angle [mMPTA], 90.7°). (B) Clinical photograph. JLCA: joint line convergence angle.

**Figure 2.**
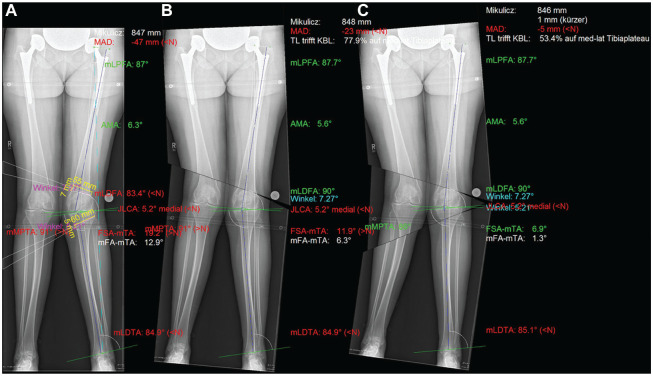
Preoperative plan for a double-level osteotomy in the same patient shown in Figure 1. Femoral medial closing-wedge distal femoral osteotomy with 7-mm wedge height and medial closing-wedge high tibial osteotomy with 5-mm wedge height (A). A postoperative alignment with the following angles was planned: mechanical lateral distal femoral angle (mLDFA) of 90°, mechanical tibiofemoral angle (mTFA) of 1.3°, and mechanical medial proximal tibial angle (mMPTA) of 86°. JLCA: joint line convergence angle.

Radiographic assessment was performed to measure several parameters, including the mechanical tibiofemoral angle (mTFA), mLDFA, mMPTA, joint line convergence angle (JLCA), posterior distal femoral angle (PDFA), and patellar height (Insall-Salvati Index,^21^ Caton-Deschamps Index,^8^ and Ihle-Schröter Index^20^) and tibial slope according to Amendola et al^3^ using a full weightbearing anteroposterior full-length radiograph and a lateral-view radiograph of the knee. We evaluated these parameters preoperatively and at the time of the postoperative follow-up. Deformity analysis and preoperative planning were conducted using mediCAD digital planning software (Hectec).^43^ The goal was to obtain the desired alignment combined with joint angles in the range of the normal values (Figures 1 and 2).

Depending on the degree of lateral and medial OA, an mTFA of 0° to –2° was intended. Surgical accuracy was calculated by subtracting the postoperatively achieved correction from the preoperatively intended correction. For this analysis, the results were mathematically converted to absolute values (positive integers). Thus, surgical accuracy was reported as deviation from the intended correction, with directionless magnitude and lower values representing greater accuracy.^44^ Preoperative knee function and postoperative outcome were evaluated using established and standardized instruments: Hospital for Special Surgery (HSS) score, Oxford Knee Score (OKS), and Lysholm score.

### Surgical Technique

**Figure 3.**
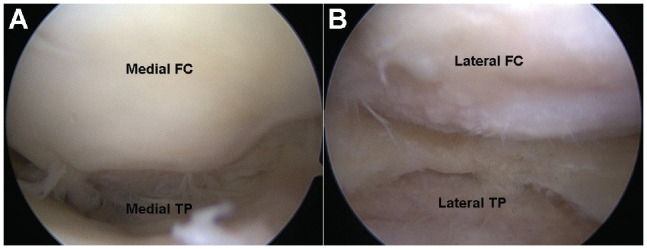
Perioperative arthroscopy showing the medial (A) and the lateral joint space (B). FC, femoral condyle; TP, tibial plateau.

In all patients, an arthroscopy was performed to evaluate the cartilage and remove the intercondylar notch osteophytes. Especially in severe OA, osteophytes can weaken the anterior cruciate ligament and cause a loss of extension (Figure 3).

**Figure 4.**
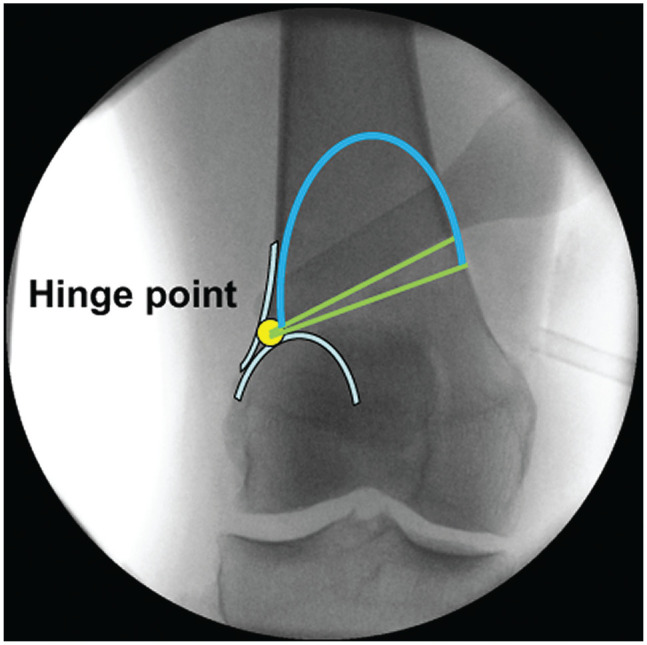
The hinge (yellow dot) is defined at the intersection of the shadow of the posterior lateral condyle and the lateral cortex. The transversal cuts are marked with green. The length of the ascending cut (blue line) should be 5 to 6 cm.

**Figure 5.**
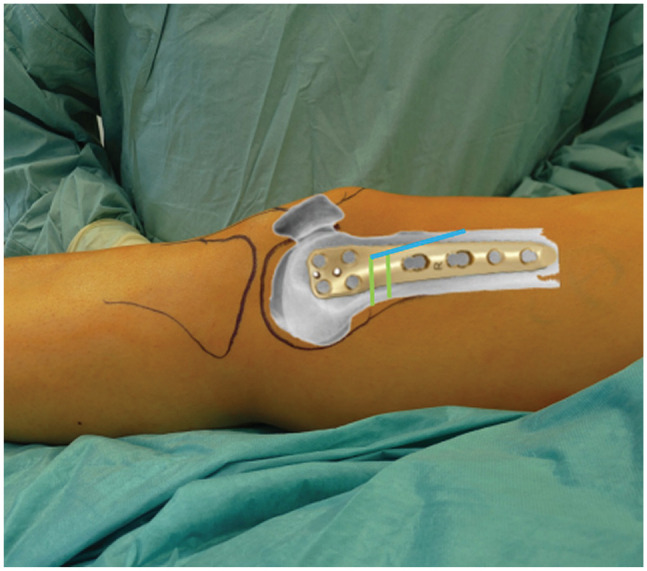
Image demonstrating the level of the double-level osteotomy and plate position in relation to the soft tissue. The blue line indicates the ascending cut, and the green lines indicate oblique transversal cuts.

After performing the arthroscopy, we started the DLO with the biplanar closing-wedge DFO using a minimally invasive approach.^28,55^ The location of the skin incision is important for a minimally invasive approach. Usually, a 5- to 6-cm skin incision at the distal medial femur is long enough. The oblique transversal bone cut is from proximal medial to distal lateral, which we recommend taking into account (Figures 4 and 5). Then, we make an incision of the fascia of the vastus medialis muscle and perform blunt preparation below the muscle belly to the bone. There are 2 possible techniques to position the radiolucent retractor posterior to the femur. The first one is to perform an incision of the intermuscular septum and bluntly dissect the soft tissue attachments from the posterior aspect of the bone. Then, the radiolucent retractor can be inserted close to the bone from medial to lateral. The second technique is to detach the periosteum along with the intermuscular septum. Then, similarly, the retractor is pushed from medial to lateral after bluntly dissecting the soft tissue of the posterior aspect of the femur. We prefer to just detach the intermuscular septum. During soft tissue preparation, the surgeon must be aware of the anatomy of the vessels. The artery is more lateral than medial at this level of the femur.^54^ Using a radiolucent retractor helps to protect the vessels, and it is still possible to use an intensifier while performing the osteotomy.

**Figure 6.**
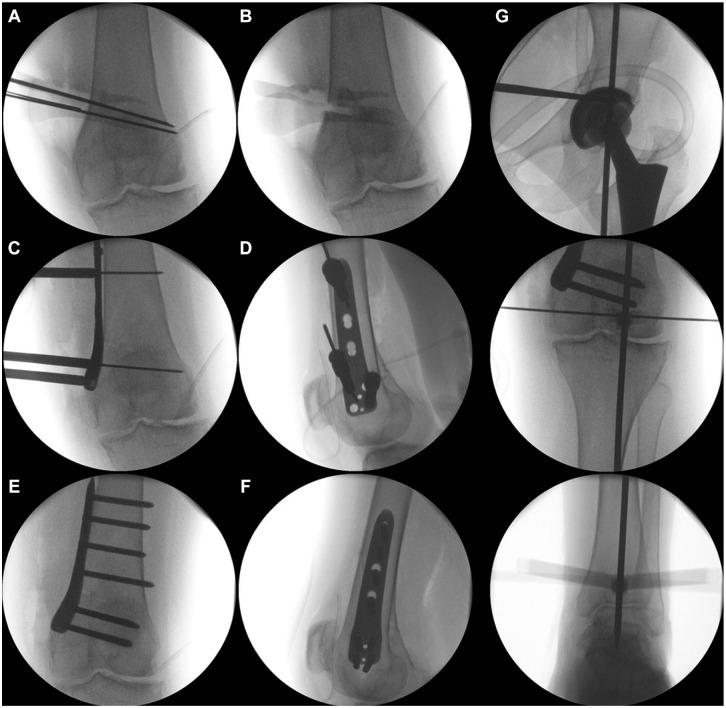
Intraoperative radiographs of the distal femoral osteotomy: positioning of the K-wires. (A) The hinge is defined as the intersection of the shadow of the posterior lateral condyle and the lateral cortex. (B) Removal of the bone wedge. (C, D) Plate position. (E, F) Radiographs of the completed distal femoral osteotomy. (G) Intraoperative control of the alignment using the alignment rod.

Afterward, 2 or 4 K-wires (depending on the experience of the surgeon) are placed according to the preoperative plan to create an isosceles triangle and a cage for the saw blade. The distance between the K-wires is the same as the height of the wedge base, which was simulated and planned preoperatively, and the hinge is defined as the intersection of the shadow of the posterior lateral condyle and lateral cortex (Figures 4 and 6A).

The tips of the K-wires on the opposite cortex should not touch each other. A distance of 1 to 2 mm between the K-wires is helpful because the saw blade is usually 1.27 mm thick. Otherwise, it will not be possible to saw close to the hinge (Figure 6A).

The oblique transversal cut should stop 0.5 cm before the lateral cortex (Figure 6B). As illustrated by the green lines in Figure 5, the oblique transversal cut is performed exclusively in the posterior two-thirds of the bone. The anterior third of the femur is not cut to prevent an anterior flange fracture (Figure 5). After finishing this cut, we make the ascending cut, which should be 5 to 6 cm in length. The minimum bone thickness of the ascending cut at the level of the oblique osteotomy is 1 cm.

**Figure 7.**
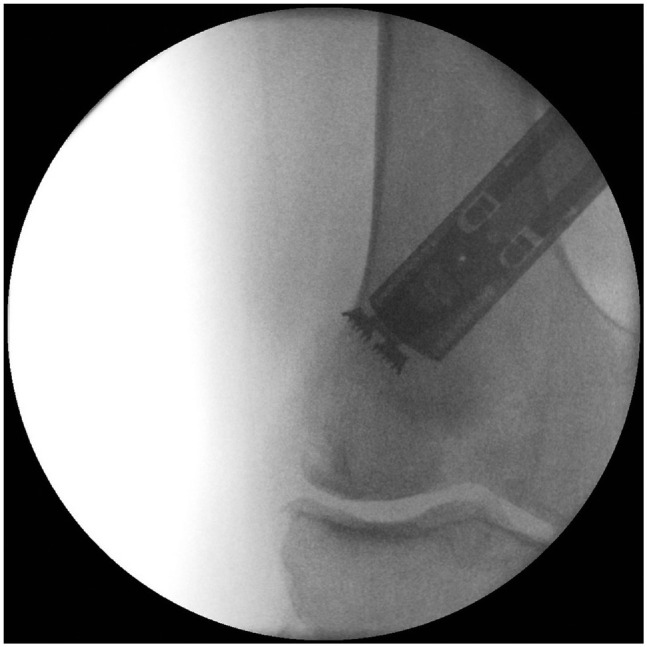
The saw blade must be rotated to cut the bone distally with the edge of the saw blade and avoid soft tissue damage at the lateral side.

The saw blade is rotated in the plane of the ascending cut in order to cut the bone distally with the edge of the saw blade (Figure 7). In the minimally invasive approach, damage to the soft tissue is less with the Precision Saw (Stryker) than with a normal oscillating saw.

The bone wedge can be removed. The osteotomy should be flexible so that it can be easily closed to avoid a hinge fracture.

**Figure 8.**
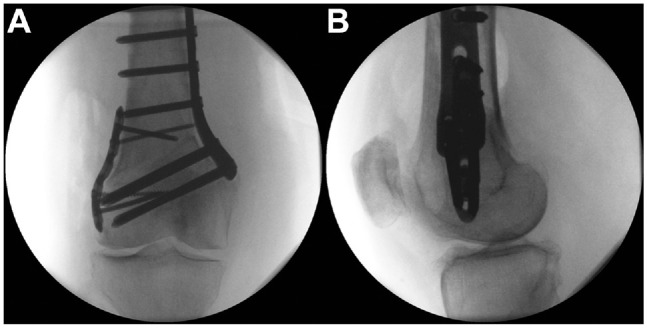
Fixation of the lateral hinge of a dislocated hinge fracture using a preshaped 5-hole, 3.5-mm locking compression plate. (A) Anteroposterior and (B) lateral radiographs.

In case of a dislocated hinge fracture, fixation of the lateral hinge must be performed. A preshaped 5-hole, 3.5-mm locking compression plate with 2 screws proximal and 2 screws distal is useful. The osteotomy is still flexible and can be closed (Figure 8). This hinge plate is effective for all distal femoral osteotomies (medial or lateral, opening or closing wedge).

The osteotomy is fixed by an angular stable plate (TomoFix MDF; DePuy Synthes) using 2 K-wires (Figure 6C). For the proximal K-wire, an additional stab incision is performed. The plate position is checked radiographically in both planes (Figure 6D). If readjustment is required, just 1 K-wire can be removed and the plate rotated. Fixation starts distally from the osteotomy with 4 screws. If this is not possible given the shape of the distal femur, bending of the plate is required. After fixation of all 4 distal screws, eccentric drilling is performed in the first proximal hole and a cortical screw inserted, leading to compression of the osteotomy. Before tightening the screw, we remove the proximal K-wire. Then, the 2 most proximal screws are inserted after a stab incision. The other 2 proximal screws can be inserted via the distal approach. The final radiographs of the closing-wedge DFO should include the alignment rod (Figure 6, E-G).

Comparison with the preoperative plan is required. For interpretation of the alignment in the supine position, it is important that the surgeon knows the ligament's stability and is aware of the risk of overcorrection in case of large ligament instability. The fascia of the muscle should be closed, a drain inserted, and the wound closed.

There are 2 possibilities for the medial HTO approach: an oblique incision^27,50^ and a straight incision.^23^ They have been previously reported for the opening-wedge HTO, but the same approaches are used for medial closing-wedge HTO. In the presented case series, just 1 case using an oblique incision was included. In all other cases, a straight incision was used. The fascia was opened in an L shape at the anterior border of the superficial medial collateral ligament (MCL), followed by preparation for insertion of the patellar tendon. In contrast to the opening-wedge HTO technique, complete detachment of the MCL must be avoided, even of the superficial MCL, because the osteotomy will be performed distal to insertion of the deep MC. Thus, there is a risk of ligament instability in midflexion of the knee if the MCL is detached completely. Use of a well-shaped retractor is recommended to avoid too much detachment and reach the lateral part of the tibia.

**Figure 9.**
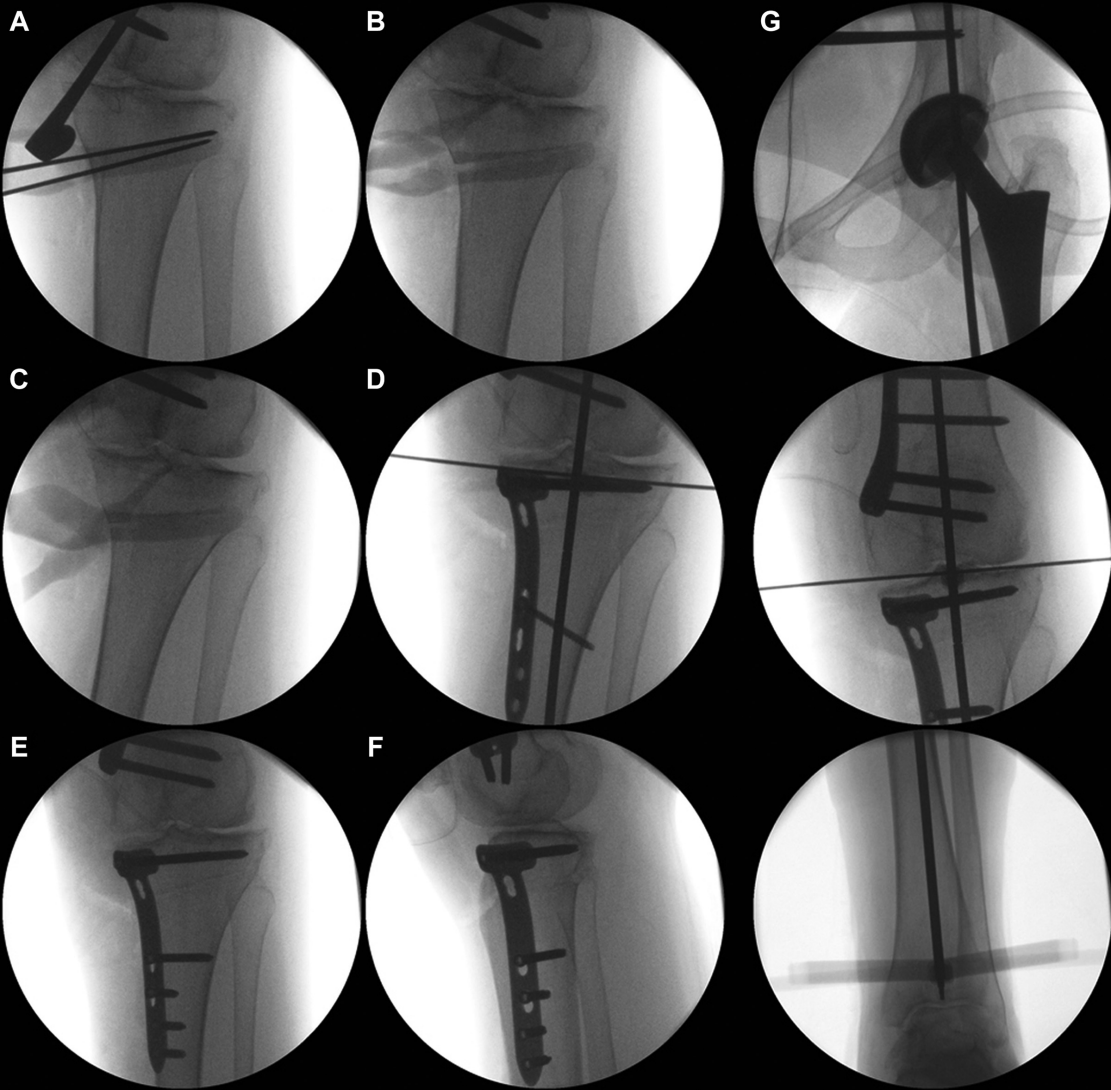
Intraoperative radiographs of the medial closing-wedge high tibial osteotomy. (A) Positioning of the K-wires aimed at the fibular head. (B, C) Removal of the bone wedge and closing of the osteotomy. Distal to the osteotomy a bicortical screw is placed via eccentric drilling. (D) After compression of the osteotomy the alignment is checked radiographically using an alignment rod. (E, F) Completed high tibial osteotomy. (G) Alignment control using the alignment rod.

**Figure 10.**
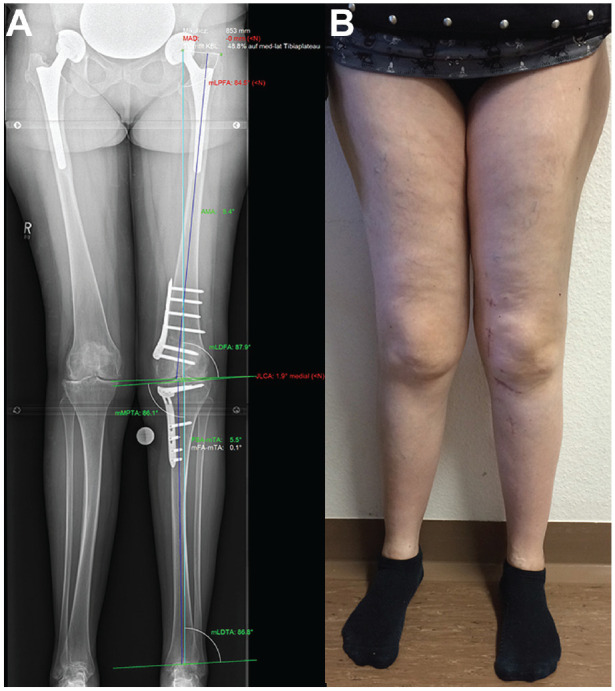
(A) Postoperative radiograph (mechanical tibiofemoral angle [mTFA], 0.1°; mechanical lateral distal femoral angle [mLDFA], 87.9°; mechanical medial proximal tibial angle [mMPTA], 86.1°) and (B) clinical photograph obtained in the same patient shown in Figure 1. JLCA: joint line convergence angle.

The main difference compared with the opening-wedge HTO is that there are 2 oblique bone cuts required, which should be performed very precisely. Otherwise, the osteotomy cannot be closed, and the risk of a hinge fracture is high. After positioning the retractor, we place 2 or 4 K-wires within the planned distance of the wedge base (Figure 9A). Both oblique transversal cuts are made first, followed by the ascending cut behind the patellar tendon (Figure 9B). Afterward, the bone wedge is removed. If the osteotomy is not flexible enough, the hinge has to be weakened using the saw. After closing the osteotomy (Figure 9C), we position a TomoFix MHT plate (DePuy Synthes) and fix it with 2 K-wires. The plate position is checked radiographically. The proximal holes of the plate are used for fixation with angular stable locking screws. Afterward, in hole 1 distal to the osteotomy, a bicortical screw is placed via eccentric drilling. After compression of the osteotomy, the alignment is checked radiographically using an alignment rod (Figure 9D). The fixation is completed by inserting 3 locking screws distally. Finally, the cortical screw is replaced by a bicortical locking screw. The final radiograph shows the correct position of the plate (Figure 9, E and F) and alignment according to the plan (Figure 9G). A drain is placed subcutaneously, and wound closure is performed. The radiographic and clinical views at the final follow-up show a straight leg (Figure 10).

### Postoperative Rehabilitation

The patients were allowed partial weightbearing of 20 kg from day 1 after the surgery. Full weightbearing was allowed after 6 weeks. Usually, it took an additional 2 weeks for patients to be able to walk without crutches. There was no limitation in range of motion. No orthosis was required. Physical therapy was recommended during the first 12 weeks of follow-up.

### Statistical Analysis

Numeric values are reported as mean ± standard deviation (range). Paired *t* tests were performed to compare pre- and postoperative radiographic and clinical outcome variables. JMP statistical software (Version 12; SAS Institute) was used to analyze the data, with the level of significance set at *P* < .05.

## Results

The preoperative and postoperative radiographic parameters are compared in Table 2. The mTFA changed significantly, from 10.3°± 3.5° preoperatively to –1.8°± 3.4° postoperatively (*P* < .001). The mean correction angle of both the femur and tibia was 12.1°± 4.5°. The mean changes in the mLDFA and mMPTA were 5.1°± 2.7° and 5.9°± 2.2°, respectively (*P* < .001 for both). The PDFA decreased significantly, from 85.9°± 3.1° to 84.2°± 2.4° (*P* < .01). There was no significant difference in pre- and postoperative JLCA, tibial slope, or patellar height.

**Table 2:** Pre- and Postoperative Radiographic Parameters^*a*^

Parameter	Preoperative	Postoperative	Difference	*P*
mTFA, deg	10.3 ± 3.5 (6.3 to 16.6)	−1.8 ± 3.4 (–7.6 to 3.5)	12.1 ± 4.5 (6.4 to 18.9)	<.001
mLDFA, deg	84.9 ± 2.6 (79.3 to 87.1)	90.0 ± 3.3 (83.4 to 94.3)	5.1 ± 2.7 (1.7 to 10.3)	<.001
mMPTA, deg	92.0 ± 1.9 (89.6 to 94.7)	86.1 ± 2.6 (80.5 to 89.3)	5.9 ± 2.2 (3.5 to 9.2)	<.001
JLCA, deg	3.3 ± 2.3 (0.3 to 6.2)	2.6 ± 2.1 (0.3 to 6.0)	0.8 ± 2.1 (–1.3 to 5.4)	NS
Patellar height				
Insall-Salvati Index	1.19 ± 0.1 (1.06 to 1.36)	1.25 ± 0.2 (0.96 to 1.67)	0.06 ± 0.16 (–0.4 to 0.12)	NS
Ihle-Schröter Index	1.45 ± 0.17 (1.27 to 1.78)	1.48 ± 0.17 (1.26 to 1.85)	0.03 ± 0.11 (–0.16 to 0.17)	NS
Caton-Deschamps Index	0.98 ± 0.17 (0.72 to 1.2)	0.98 ± 0.22 (0.69 to 1.34)	0.00 ± 0.15 (–0.18 to 0.26)	NS
PDFA	85.9 ± 3.1 (80.0 to 89.0)	84.2 ± 2.4 (80.3 to 87.9)	1.6 ± 1.5 (–1.3 to 3.1)	<.01
Tibial slope, deg^*b*^	5.4 ± 2.4 (1.5 to 9.4)	3.8 ± 3.1 (0.7 to 9.5)	1.6 ± 2.9 (–3.8 to 5.6)	NS

a Data are reported as mean ± SD (range). JLCA, joint line convergence angle; mLDFA, mechanical lateral distal femoral angle; mMPTA, mechanical medial proximal tibial angle; mTFA, mechanical tibiofemoral angle; NS, not significant; PDFA, posterior distal femoral angle.

b Measured according to Amendola et al.^3^

The accuracy of the correction was high (Table 3), especially the mean corrections for mTFA, mMPTA, and mLDFA, which were all <3°. Despite this result, the range of accuracy was very narrow for mTFA (0.6°-6.6°) and mMPTA (0.1°-3.2°), whereas for mLDFA the was wider (0°-6.5°). Comparison of pre- with postoperative clinical scores showed significant improvement in all 3 scores (*P* < .001 for all) (Table 4). The preoperative, planned, and postoperative radiological alignment parameters as well as the OKS and Lysholm score for each included knee are presented in Table 5.

**Table 3:** Accuracy of Radiographic Parameters^*a*^

Parameter	Planned	Postoperative	Accuracy
mTFA, deg	−0.5 ± 0.6 (1.0 to 0.5)	−1.8 ± 3.4 (–7.6 to 3.5)	2.7 ± 1.9 (0.6 to 6.6)
mLDFA, deg	90.1 ± 0.8 (89.9 to 92.0)	90.0 ± 3.3 (83.4 to 94.3)	2.1 ± 2.1 (0 to 6.5)
mMPTA, deg	86.9 ± 2.0 (83.7 to 89.4)	86.1 ± 2.6 (80.5 to 89.3)	1.6 ± 0.9 (0.1 to 3.2)

a Data are reported as mean ± SD (range). mLDFA, mechanical lateral distal femoral angle; mMPTA, mechanical medial proximal tibial angle; mTFA, mechanical tibiofemoral angle.

**Table 4:** Pre- and Postoperative Clinical Scores^*a*^

Score	Preoperative	Postoperative	Difference	*P*
HSS	67 ± 11 (54-84)	93 ± 4 (85-98)	26 ± 10 (12-39)	<.001
OKS	29 ± 7 (20-39)	43 ± 3 (40-48)	14 ± 5 (9-23)	<.001
Lysholm	41 ± 24 (18-75)	89 ± 8 (77-100)	48 ± 18 (25-72)	<.001

a Data are reported as mean ± SD (range). HSS, Hospital for Special Surgery; OKS, Oxford Knee Score.

**Table 5:** Breakdown of Radiographic Alignment Parameters and Clinical Scores by Operated Knee (n = 9)^*a*^

Knee	mTFA, deg			mLDFA, deg			mMPTA, deg			OKS		Lysholm	
	Pre	Planned	Post	Pre	Planned	Post	Pre	Planned	Post	Pre	Post	Pre	Post
1	13.4	−0.5	−5.5	83.3	92	93.6	91	86	83.8	28	42	53	85
2	8.4	−0.6	−3	87.1	90.3	88.8	94.4	88.5	86.2	37	47	73	100
3	12.7	0.5	3.5	82.9	90	87.9	91	85.6	86.7	23	40	22	83
4	6.3	0	−0.6	87.1	90	89.5	92.8	89.4	89.3	39	48	75	100
5	7.5	−1	1.1	86.8	91	91	89.6	85.3	86	35	44	58	95
6	6.6	−1	−7.6	86	90	94.3	93	89.3	87.9	27	45	24	77
7	16.6	0	−2.1	79.3	89.9	83.4	89.7	83.7	80.5	20	43	18	90
8	10.3	−1	−2.4	86.7	91.5	92.2	94.7	88.1	86.6	23	40	22	83
9	11	−1	0.5	85.3	91	89.4	92.1	85.8	87.8	29	42	22	90

a mLDFA, mechanical lateral distal femoral angle; mMPTA, mechanical medial proximal tibial angle; mTFA, mechanical tibiofemoral angle; OKS, Oxford Knee Score; Pre, preoperative; Post, postoperative.

Regarding complications, 1 patient had an intraoperative lateral hinge fracture, which was fixed using a hinge plate, as mentioned above. The healing was uneventful, and the OKS, HSS, and Lysholm scores improved from 20 to 43, from 68 to 85, and from 18 to 90, respectively. These results were comparable to those in the other patients. Therefore, the hinge fracture had no negative influence on the outcome. However, the surgical time was much longer. Another patient experienced blunt trauma at the medial proximal tibia 3 months after the surgery, and a few days later, the hematoma became infected (*Staphylococcus aureus*). Immediate removal of the implants was possible because of completed bone healing. The final OKS, HSS, and Lysholm scores were 44, 97, and 95, respectively.

## Discussion

The most important finding of this study was that the clinical scores improved significantly after varus DLO in moderate-to-severe valgus knees with lateral OA. Moreover, the surgical accuracy was high. To the best of our knowledge, this is the first study to report the outcomes of DLO combining closing-wedge DFO and closing-wedge HTO for valgus knees.

Several studies have reported the results of medial closing-wedge DFO for valgus knees.^**^ Finkelstein et al^15^ found a 10-year survival rate of 64%, while Backstein et al^5^ reported a 10-year survival rate of 82% and a 15-year survival rate of 45%. Kosashvili et al^25^ showed deterioration of knee function at the 10-year follow-up, but function remained much better than before surgery. Meanwhile, the results of medial closing-wedge HTO for valgus knees were relatively worse compared with those of medial opening-wedge HTO for varus knees.^11,48^ Researchers have also reported that the excessive joint line obliquity caused by huge correction on the tibia was associated with inferior results. Eberbach et al^12^ showed that the valgus deformity is located in the femur (23.6%), tibia (41.0%), or femur and tibia combined (26.9%). Therefore, precise assessment of the deformity and detailed planning of the surgery is needed. The present study showed satisfying results after varus DLO in valgus knees. If excessive joint line obliquity could be produced by a single-level correction (the femur alone or tibia alone), we suggest consideration of a DLO.

The optimal postoperative alignment has been controversial in the correction of valgus deformity, including the joint line obliquity. Some researchers have recommended correction of the aTFA to 6° to 10°,^15,32,56^ and others have recommended an mTFA between 0° and 3°.^6,30^ Marin Morales et al^29^ suggested that appropriate correction was essential to obtain the best clinical results in the valgus deformity, and if undercorrection (>5° of valgus) or overcorrection (>5° of varus) was achieved, failure was often observed. Schröter et al^46^ reported that a postoperative mLDFA >90° resulted in an inferior clinical outcome compared with mLDFA >85° to 90° and noted that joint line obliquity should be avoided in the correction of valgus deformity. In the present study, we planned the postoperative mTFA to 0° to 1° varus, and the postoperative mTFA was within 3° of the planning degrees in 7 knees (77.8%). Although there were 2 cases of overcorrection, the mMPTA was <10° in all the cases. Coventry^11^ reported that obliquity of as much as 10° was found to be compatible with a good result, and also found that obliquity >10°, although statistically nonsignificant, was associated with a poor result. It might be one of the reasons why all the cases in the current study improved.

Regarding total knee replacement after varus DFO, relatively good results have been reported.^7,15,35^ However, some technical difficulties can be encountered during the surgery. Nelson et al^35^ mentioned that extra-articular varus deformity of the femur often results in a situation in which the femoral anatomic axis intersects the lateral femoral condyle rather than the intercondylar notch, and determination of the appropriate location of the starting hole using an intramedullary guide is important for obtaining optimal femoral alignment in these cases. They also cautioned that resection of relatively more bone from the distal aspect of the lateral femoral condyle than from the distal aspect of the medial femoral condyle is often necessary. As a result, lateral ligamentous instability that is not correctable using standard medial releases can be observed, which requires constrained prostheses. It might occur more frequently if large corrections were performed at only 1 level. Therefore, we suggest that surgeons should consider DLO if the preoperative plan would result in unphysiological angles with a single-level osteotomy.

### Limitations

Our present study has some limitations. First, the sample size was small. We preselected patients strictly indicated for surgery. Second, there was no control group in our study. The presence of a control group would have enhanced the value of the results. However, a control group was deemed ethically unjustifiable given the retrospective design of the study. Finally, the follow-up period was relatively short, at 25.1 ± 11.1 months. Mid- and long-term outcomes should be reported in the future.

## Conclusion

The results of the present study demonstrated that patients who underwent varus DLO for valgus knee showed improved knee function in the short-term follow-up. Varus DLO can be a surgical option to restore optimal alignment and joint line obliquity in patients with moderate-to-severe valgus malalignment.

## References

[1] Aglietti P Menchetti PP . Distal femoral varus osteotomy in the valgus osteoarthritic knee. Am J Knee Surg. 2000;13(2):89-95. 11281336

[2] Alves P van Rooij F Kuratle T Saffarini M Miozzari H . Consistent indications, targets and techniques for double-level osteotomy of the knee: a systematic review. Knee Surg Sports Traumatol Arthrosc. 2022;30(12):4078-4087. 35290484 10.1007/s00167-022-06915-6

[3] Amendola A Rorabeck CH Bourne RB Apyan PM . Total knee arthroplasty following high tibial osteotomy for osteoarthritis. J Arthroplasty. 1989;4(suppl):S11-S17. 2584982 10.1016/s0883-5403(89)80002-6

[4] Babis GC An KN Chao EY Rand JA Sim FH . Double level osteotomy of the knee: a method to retain joint-line obliquity. Clinical results. J Bone Joint Surg Am. 2002;84(8):1380-1388. 12177268 10.2106/00004623-200208000-00013

[5] Backstein D Morag G Hanna S Safir O Gross A . Long-term follow-up of distal femoral varus osteotomy of the knee. J Arthroplasty. 2007;22(4 suppl 1):2-6. 10.1016/j.arth.2007.01.026 17570268

[6] Cameron HU Botsford DJ Park YS . Prognostic factors in the outcome of supracondylar femoral osteotomy for lateral compartment osteoarthritis of the knee. Can J Surg. 1997;40(2):114-118. 9126124 PMC3952972

[7] Cameron HU Park YS . Total knee replacement following high tibial osteotomy and unicompartmental knee. Orthopedics. 1996;19(9):807-808. 8887430 10.3928/0147-7447-19960901-30

[8] Caton J Deschamps G Chambat P Lerat JL Dejour H . Patella infera. Apropos of 128 cases. [Article in French]. Rev Chir Orthop Reparatrice Appar Mot. 1982;68(5):317-325. 6216535

[9] Cerciello S Lustig S Servien E Batailler C Neyret P . Correction of tibial valgus deformity. J Knee Surg. 2017;30(5):421-425. 28575907 10.1055/s-0037-1603504

[10] Chahla J Mitchell JJ Liechti DJ , et al. Opening- and closing-wedge distal femoral osteotomy: a systematic review of outcomes for isolated lateral compartment osteoarthritis. Orthop J Sports Med. 2016;4(6):2325967116649901. 27331074 10.1177/2325967116649901 PMC4900333

[11] Coventry MB . Proximal tibial varus osteotomy for osteoarthritis of the lateral compartment of the knee. J Bone Joint Surg Am. 1987;69(1):32-38. 3805069

[12] Eberbach H Mehl J Feucht MJ , et al. Geometry of the valgus knee: contradicting the dogma of a femoral-based deformity. Am J Sports Med. 2017;45(4):909-914. 28125900 10.1177/0363546516676266

[13] Elson DW . The surgical accuracy of knee osteotomy. Knee. 2017;24(2):167-169. 28317593 10.1016/j.knee.2017.02.008

[14] Elson DW Petheram TG Dawson MJ . High reliability in digital planning of medial opening wedge high tibial osteotomy, using Miniaci's method. Knee Surg Sports Traumatol Arthrosc. 2015;23(7):2041-2048. 24584646 10.1007/s00167-014-2920-x

[15] Finkelstein JA Gross AE Davis A . Varus osteotomy of the distal part of the femur. A survivorship analysis. J Bone Joint Surg Am. 1996;78(9):1348-1352. 8816649 10.2106/00004623-199609000-00008

[16] Forkel P Achtnich A Metzlaff S Zantop T Petersen W . Midterm results following medial closed wedge distal femoral osteotomy stabilized with a locking internal fixation device. Knee Surg Sports Traumatol Arthrosc. 2015;23(7):2061-2067. 24676790 10.1007/s00167-014-2953-1

[17] Haviv B Bronak S Thein R Thein R . The results of corrective osteotomy for valgus arthritic knees. Knee Surg Sports Traumatol Arthrosc. 2013;21(1):49-56. 22940779 10.1007/s00167-012-2180-6

[18] Healy WL Anglen JO Wasilewski SA Krackow KA . Distal femoral varus osteotomy. J Bone Joint Surg Am. 1988;70(1):102-109. 3335557

[19] Hofmann S Lobenhoffer P Staubli A Van Heerwaarden R . Osteotomies of the knee joint in patients with monocompartmental arthritis. [Article in German]. Orthopade. 2009;38(8):755-769. 19629433 10.1007/s00132-009-1458-y

[20] Ihle C Ahrend M Grunwald L , et al. No change in patellar height following open wedge high tibial osteotomy using a novel femur-referenced measurement method. Knee. 2017;24(5):1118-1128. 28673604 10.1016/j.knee.2017.06.006

[21] Insall J Salvati E . Patella position in the normal knee joint. Radiology. 1971;101(1):101-104. 5111961 10.1148/101.1.101

[22] Jackson JP Waugh W . The technique and complications of upper tibial osteotomy. A review of 226 operations. J Bone Joint Surg Br. 1974;56(2):236-245. 4855112

[23] Kfuri M Lobenhoffer P . High tibial osteotomy for the correction of varus knee deformity. J Knee Surg. 2017;30(5):409-420. 28591931 10.1055/s-0037-1603757

[24] Kim JH Kim HJ Lee DH . Survival of opening versus closing wedge high tibial osteotomy: a meta-analysis. Sci Rep. 2017;7(1):7296. 28779084 10.1038/s41598-017-07856-8 PMC5544741

[25] Kosashvili Y Safir O Gross A , et al. Distal femoral varus osteotomy for lateral osteoarthritis of the knee: a minimum ten-year follow-up. Int Orthop. 2010;34(2):249-254. 19468727 10.1007/s00264-009-0807-0 PMC2899359

[26] Learmonth ID . A simple technique for varus supracondylar osteotomy in genu valgum. J Bone Joint Surg Br. 1990;72(2):235-237. 2312562 10.1302/0301-620X.72B2.2312562

[27] Lobenhoffer P Agneskirchner JD . Improvements in surgical technique of valgus high tibial osteotomy. Knee Surg Sports Traumatol Arthrosc. 2003;11(3):132-138. 12774149 10.1007/s00167-002-0334-7

[28] Lobenhoffer P Kley K Freiling D van Heerwaarden R . Distale Femurosteotomie in schließender biplanarer Technik mit Stabilisierung durch spezifischen Plattenfixateur. Oper Orthop Traumatol. 2017;29(4):306-319. 28497247 10.1007/s00064-017-0493-9

[29] Marin Morales L Gomez Navalon L Salido Valle J . Treatment of osteoarthritis of the knee with valgus deformity by means of varus osteotomy. Acta Orthop Belg. 2000;66(3):272-278. 11033918

[30] Marti RK Verhagen RA Kerkhoffs GM Moojen TM . Proximal tibial varus osteotomy. Indications, technique, and five to twenty-one-year results. J Bone Joint Surg Am. 2001;83(2):164-170. 11216676

[31] Mathews J Cobb AG Richardson S Bentley G . Distal femoral osteotomy for lateral compartment osteoarthritis of the knee. Orthopedics. 1998;21(4):437-440. 9571677 10.3928/0147-7447-19980401-08

[32] McDermott AG Finklestein JA Farine I , et al. Distal femoral varus osteotomy for valgus deformity of the knee. J Bone Joint Surg Am. 1988;70(1):110-116. 3335559

[33] Nakayama H Schröter S Yamamoto C , et al. Large correction in opening wedge high tibial osteotomy with resultant joint-line obliquity induces excessive shear stress on the articular cartilage. Knee Surg Sports Traumatol Arthrosc. 2018;26(6):1873-1878. 28831525 10.1007/s00167-017-4680-x

[34] Nakayama H Iseki T Kanto R , et al. Physiologic knee joint alignment and orientation can be restored by the minimally invasive double level osteotomy for osteoarthritic knees with severe varus deformity. Knee Surg Sports Traumatol Arthrosc. 2020;28(3):742-750. 30196434 10.1007/s00167-018-5103-3

[35] Nelson CL Saleh KJ Kassim RA , et al. Total knee arthroplasty after varus osteotomy of the distal part of the femur. J Bone Joint Surg Am. 2003;85(6):1062-1065. 12784003 10.2106/00004623-200306000-00012

[36] Saithna A Kundra R Modi CS Getgood A Spalding T . Distal femoral varus osteotomy for lateral compartment osteoarthritis in the valgus knee. A systematic review of the literature. Open Orthop J. 2012;6:313-319. 22905074 10.2174/1874325001206010313 PMC3419938

[37] Saito T Kumagai K Akamatsu Y Kobayashi H Kusayama Y . Five- to ten-year outcome following medial opening-wedge high tibial osteotomy with rigid plate fixation in combination with an artificial bone substitute. Bone Joint J. 2014;96(3):339-344. 24589788 10.1302/0301-620X.96B3.32525

[38] Saragaglia D Chedal-Bornu B . Computer-assisted osteotomy for valgus knees: medium-term results of 29 cases. Orthop Traumatol Surg Res. 2014;100(5):527-530. 25087004 10.1016/j.otsr.2014.04.002

[39] Saragaglia D Mercier N Colle PE . Computer-assisted osteotomies for genu varum deformity: which osteotomy for which varus? Int Orthop. 2010;34(2):185-190. 19305996 10.1007/s00264-009-0757-6 PMC2899360

[40] Saragaglia D Nemer C Colle PE . Computer-assisted double level osteotomy for severe genu varum. Sports Med Arthrosc. 2008;16(2):91-96. 18480728 10.1097/JSA.0b013e318172b562

[41] Saragaglia D Rubens-Duval B Chaussard C . Computer-assisted combined femoral and tibial osteotomy for severe genu varum: early results in 16 patients. [Article in French]. Rev Chir Orthop Reparatrice Appar Mot. 2007;93(4):351-356. 17646816 10.1016/s0035-1040(07)90276-7

[42] Schenke M Dickschas J Simon M Strecker W . Corrective osteotomies of the lower limb show a low intra- and perioperative complication rate—an analysis of 1003 patients. Knee Surg Sports Traumatol Arthrosc. 2018;26(6):1867-1872. 28493074 10.1007/s00167-017-4566-y

[43] Schröter S Elson DW Ateschrang A , et al. Lower limb deformity analysis and the planning of an osteotomy. J Knee Surg. 2017;30(5):393-408. 28599326 10.1055/s-0037-1603503

[44] Schröter S Ihle C Elson DW , et al. Surgical accuracy in high tibial osteotomy: coronal equivalence of computer navigation and gap measurement. Knee Surg Sports Traumatol Arthrosc. 2016;24(11):3410-3417. 26801783 10.1007/s00167-016-3983-7

[45] Schröter S Ihle C Mueller J , et al. Digital planning of high tibial osteotomy. Interrater reliability by using two different software. Knee Surg Sports Traumatol Arthrosc. 2013;21(1):189-196. 22773064 10.1007/s00167-012-2114-3

[46] Schröter S Konrads C Maiotti M , et al. In closed wedge distal femur osteotomies for correction of valgus malalignment overcorrection of mLDFA should be avoided. Knee Surg Sports Traumatol Arthrosc. 2023;31(9):3992-3999. 37149824 10.1007/s00167-023-07449-1

[47] Schröter S Mueller J van Heerwaarden R , et al. Return to work and clinical outcome after open wedge HTO. Knee Surg Sports Traumatol Arthrosc. 2013;21(1):213-219. 22810885 10.1007/s00167-012-2129-9

[48] Shoji H Insall J . High tibial osteotomy for osteoarthritis of the knee with valgus deformity. J Bone Joint Surg Am. 1973;55(5):963-973. 4760103

[49] Stahelin T Hardegger F . Incomplete, supracondylar femur osteotomy. A minimally invasive compression osteosynthesis with soft implant. [Article in German]. Orthopade. 2004;33(2):178-184. 14872309 10.1007/s00132-003-0589-9

[50] Staubli AE De Simoni C Babst R Lobenhoffer P . TomoFix: a new LCP-concept for open wedge osteotomy of the medial proximal tibia—early results in 92 cases. Injury. 2003;34(suppl 2):B55-B62. 14580986 10.1016/j.injury.2003.09.025

[51] Sternheim A Garbedian S Backstein D . Distal femoral varus osteotomy: unloading the lateral compartment: long-term follow-up of 45 medial closing wedge osteotomies. Orthopedics. 2011;34(9):e488-e490. 21902140 10.3928/01477447-20110714-37

[52] Terauchi M Shirakura K Katayama M , et al. Varus inclination of the distal femur and high tibial osteotomy. J Bone Joint Surg Br. 2002;84(2):223-226. 11922364 10.1302/0301-620x.84b2.12136

[53] Thein R Bronak S Thein R Haviv B . Distal femoral osteotomy for valgus arthritic knees. J Orthop Sci. 2012;17(6):745-749. 22868701 10.1007/s00776-012-0273-1

[54] van der Woude JA van Heerwaarden RJ Bleys RL . Periosteal vascularization of the distal femur in relation to distal femoral osteotomies: a cadaveric study. J Exp Orthop. 2016;3(1):6. 26915006 10.1186/s40634-016-0042-8 PMC4735087

[55] van Heerwaarden R Brinkman JM Pronk Y . Correction of femoral valgus deformity. J Knee Surg. 2017;30(8):746-755. 28476063 10.1055/s-0037-1602138

[56] Wang JW Hsu CC . Distal femoral varus osteotomy for osteoarthritis of the knee. J Bone Joint Surg Am. 2005;87(1):127-133. 15634823 10.2106/JBJS.C.01559

[57] Zarrouk A Bouzidi R Karray B , et al. Distal femoral varus osteotomy outcome: Is associated femoropatellar osteoarthritis consequential? Orthop Traumatol Surg Res. 2010;96(6):632-636. 20829143 10.1016/j.otsr.2010.04.009

